# N-cadherin is differentially expressed in histological subtypes of papillary renal cell carcinoma

**DOI:** 10.1186/1746-1596-7-95

**Published:** 2012-08-13

**Authors:** Carl Ludwig Behnes, Bernhard Hemmerlein, Arne Strauss, Heinz-Joachim Radzun, Felix Bremmer

**Affiliations:** 1 Department of Pathology, University of Göttingen, Robert-Koch-Str. 40, 37083, Göttingen, Germany; 2 Department of Urology, University of Göttingen, Robert-Koch-Straße 40, 37075, Göttingen, Germany

**Keywords:** N-cadherin, Histological subtypes, Papillary renal cell carcinoma (RCC), Immunohistochemistry

## Abstract

**Background:**

Papillary renal cell carcinoma (RCC) represents a rare tumor, which is divided, based on histological criteria, into two subtypes. In contrast to type I papillary RCC type II papillary RCC shows a worse prognosis. So far, reliable immunohistochemical markers for the distinction of these subtypes are not available.

**Methods:**

In the present study the expression of N(neural)-, E(epithelial)-, P(placental)-, und KSP(kidney specific)-cadherin was examined in 22 papillary RCC of histological type I and 18 papillary RCC of histological type II (n = 40).

**Results:**

All papillary RCC type II displayed a membranous expression for N-cadherin, whereas type I did not show any membranous positivity for N-cadherin. E-cadherin exhibited a stronger, but not significant, membranous as well as cytoplasmic expression in type II than in type I papillary RCC. A diagnostic relevant expression of P- and KSP-cadherin could not be demonstrated in both tumor entities.

**Conclusion:**

Thus N-cadherin represents the first immunhistochemical marker for a clear cut differentiation between papillary RCC type I and type II and could be a target for therapy and diagnostic in the future.

**Virtual slides:**

The virtual slide(s) for this article can be found here: http://www.diagnosticpathology.diagnomx.eu/vs/2011556982761733

## Introduction

Renal cell carcinoma (RCC) represents a rather rare cancer with about 71,000 newly diagnosed cases per year in Europe. Approximately 31,000 of these patients die because of RCC [1]. The RCCs are devided in different histological subtypes, of which the group of papillary RCC compromise less than 10% of all RCC [2]. Based on histological criterias type I and type II papillary RCCs can be distinguished: Type I papillary RCCs show papillae covered by a single layer of cuboidal cells with a small cytoplasmic rim; furthermore, type I tumors are often infiltrated by numerous foamy macrophages. Type II tumors also form papillae covered by a monolayer of tumor cells; in contrast to type I papillary RCC these tumor cells display higher nuclear polymorphism with pseudostratification and abundant mostly eosinophilic cytoplasm; foamy macrophages, however, are rarely seen in type II papillary RCCs [3]. As typical chromosomal changes the loss of Y chromosome or significantly higher numbers of gains of 7p, 17p, and 17q were demonstrated in papillary RCCs [4,5]. Klatte et al. [6] could show a loss of 1p, loss of 3p, and a gain of 5q exclusively in type II papillary RCC by cytogenetic analyses. The subdivision of papillary RCC into two subtypes is important, because type II papillary RCC shows a shorter survival rate because of its higher grade of malignancy and progressed stage at the time of diagnosis [7-9].

Cadherins are transmembrane glycoproteins and play a role in Ca^2+^-dependent cell-cell contacts especially in adherent junctions and in the development of different organs [10,11]. They are also involved in genesis of tumors and act as metastasis suppressing proteins [12]. A decreased cadherin expression is normally found in cancers and is associated with increased metastatic potential. This could be shown in breast cancer for the extensively studied E-cadherin [13]. Current investigations showed a worse prognosis for tumors with a non tissue specific cadherin expression [14]. In the present study the expression of N-, E-, P-, and KSP-cadherin in both subtypes of papillary RCC were examined in order to find diagnostically relevant differences.

## Methods

### Tissue samples

Tumor-tissue of radical or partial nephrectomy specimens from 40 patients suffering from papillary RCC were included in this study and analysed for the expression of N-, E-, P- und KSP-cadherin. All tumors were classified in papillary RCC subtypes and staged on the basis of WHO classification [15]. Clinical and histopathological data are summarized in Table 1.

**Table 1:** Clinical and pathological data of analysed cases

	**papRCC subtype I**	**papRCC subtype II**
*n* (men/women)	22 (18/4)	18 (9/9)
mean age (years)	69,72	71,38
,7 T1 (a/b)	13(7/6)	4(3/1)
*n*T2	7	1
*n*T3 (a/b/c)	2(1/1/0)	*11(6/4/1)*
*n*T4	0	2
*n*Gl	6	0
*n*G2	15	13
*nG3*	1	5
*n*Ml	0/22	2/18
*n*Nl	1/22	1/18
*n*N2	0/22	5/18

### Immunohistochemistry

Primary immunohistochemical reactions for the analysed cadherins were performed on paraffin-embedded sections of papillary RCC as listed in Table 2. Thereafter sections were incubated with a horseradish peroxidase (HRP)-conjugated polymer consisting of antibodies to rabbit and mouse immunoglobulins (EnVision/HRP, Dako, Hamburg, Germany). Specific binding was visualized with 3,3_- diaminobenzidine (DAB; Dako). All samples were counterstained with Meyer’s haematoxylin, mounted in Super Mount Medium, and analysed by light microscopy.

**Table 2:** Applied antibodies and conditions for primary immunohistochemical reaction

	**N-Cadherin**	**E-Cadherin**	**P-Cadherin**	**KSP-Cadherin**
Clone	6 G11 (mouse)	NCH-38 (mouse)	56C1 (mouse)	MRQ-33 (mouse)
Source	Dako, Hamburg	Dako, Hamburg	Linaris, Dossenheim	Zytomed Systems, Berlin
	Germany	Germany	Germany	Germany
Pretreatment	citrate buffer	citrate buffer	citrate buffer	citrate buffer
	pH6.0 40 min	pH6.0 40 min	pH6.0 40 min	pH6.0 40 min
Dilution and	1:50 30 min RT	1:50 30 min RT	1:100 30 min RT	1:50 30 min RT
Incubation				

*RT*: room temperature.

All sections were evaluated by two independent investigators for membranous and cytoplasmic staining using the immunoreactive staining score (IRS). To establish the IRS, the percentage of positive-stained cells was evaluated first using a 0–5 scoring system: 0% of positive cells resulted in a score of 0, less than 1% in a score of 1, 1–10% in a score of 2, 10–33% in a score of 3, 33–66% in a score of 4 and 66–100% in a score of 5. Staining intensity was evaluated by a gradual scale (0, negative; 1, weak; 2, intermediate; 3, strong). For the final score the scores of intensity and of positive tumor cells were added and the mean value was calculated.

### Statistical analysis

For statistical analyses the IRS was compared between papRCC subtypes using the Wilcoxon test (GraphPad Software, SanDiego, CA, USA). A P value of < 0.05 was considered to show a significant difference. All data are presented as mean ± standard error of the mean (SEM).

## Results

**Figure 1:**
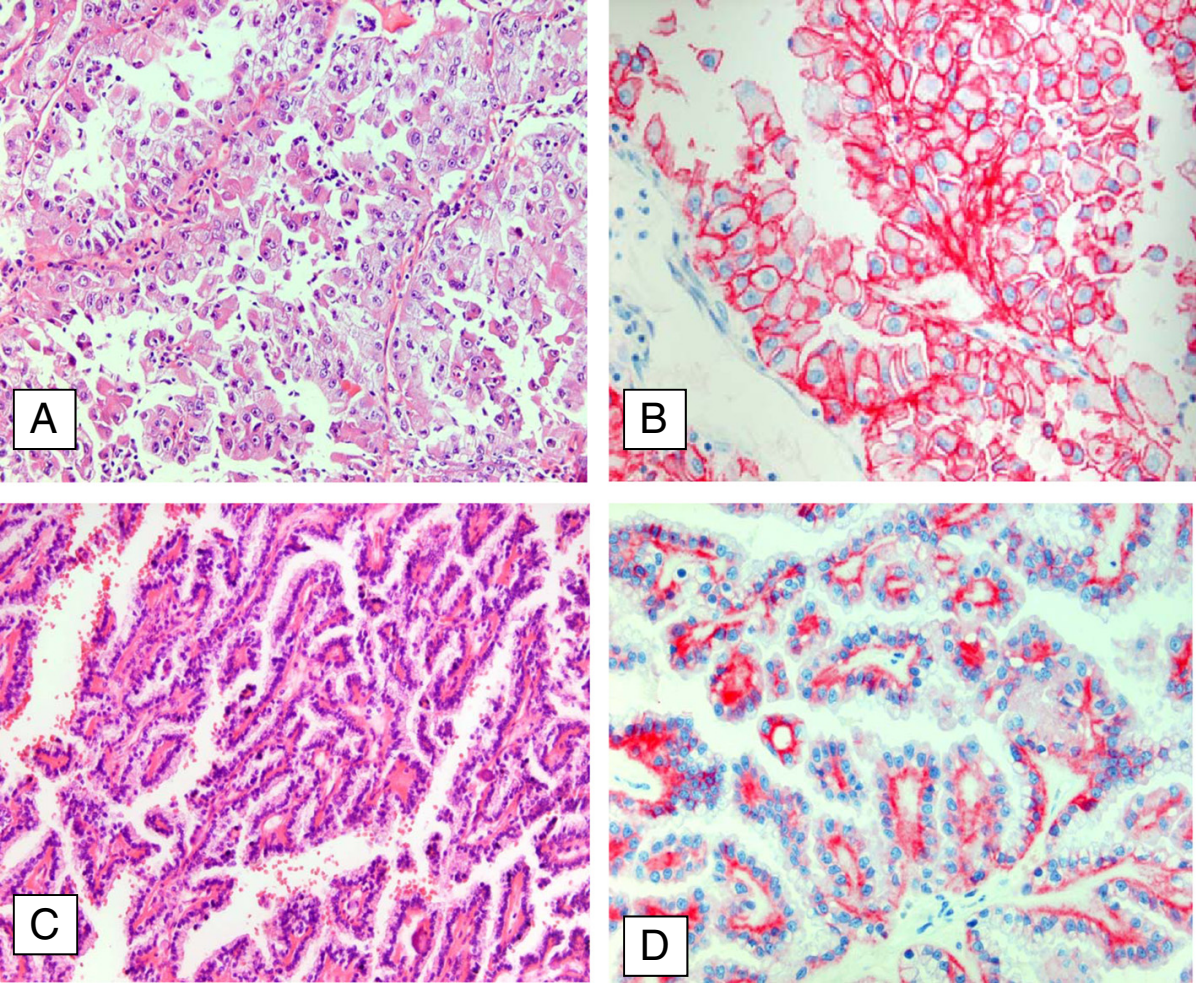
**Immunohistochemistry of N-cadherin expression in papillary RCC.** Papillary RCC type II with high nuclear polymorphism and abundant cytoplasm of the tumor cells (**A**, x20) displays a strong membranous expression of N-cadherin (**B**, x40). Papillary RCC type I with a single layer of cuboidal cells and a small cytoplasmic rim (**C**, x20) shows only a weak cytoplasmic N-cadherin (**D**, x40).

**Figure 2:**
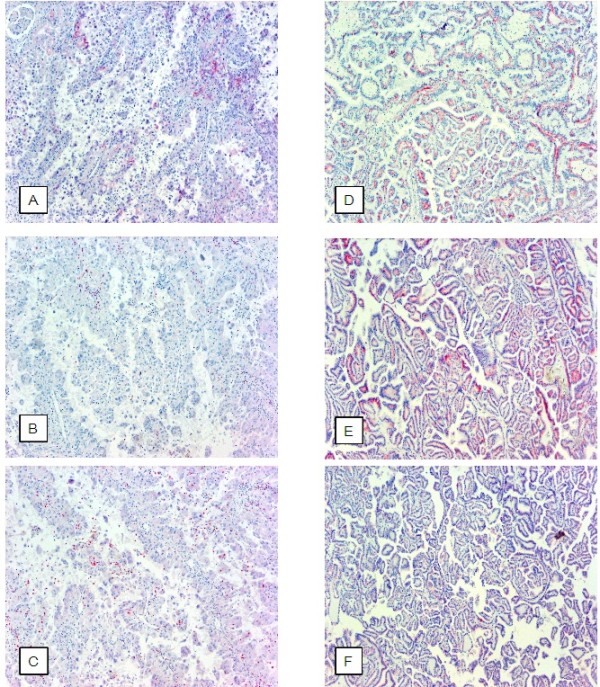
**Histopathological evaluation of cadherin expression in both sybtypes of papillary RCC.** N-cadherin showed a significant difference in membranous and cytoplasmic expression between both subtypes of papillary RCC. A significant difference for E-, P- and KSP-cadherin expression could not be demonstrated.

Immunohistochemical examinations revealed a membranous positivity for N-cadherin in all papillary RCC type II (IRS 6.28 ± 1.57), whereas type I did not show any membranous positivity for N-cadherin (IRS 0). In contrast to the membranous N-cadherin expression the cytoplasmic expression of N-cadherin showed a higher score in papillary RCC type I (IRS 5.68 ± 1.76) than in type II (IRS 2.5 ± 2.0); cytoplasmic N-cadherin was particularly detectable in the apical and basolateral parts of the tumorcells. The comparison between both subtypes of papillary RCC for cytoplasmic N-cadherin showed a significant difference (P < 0.0032) (Figure 1A-D and Figure 2).

**Figure 3:**
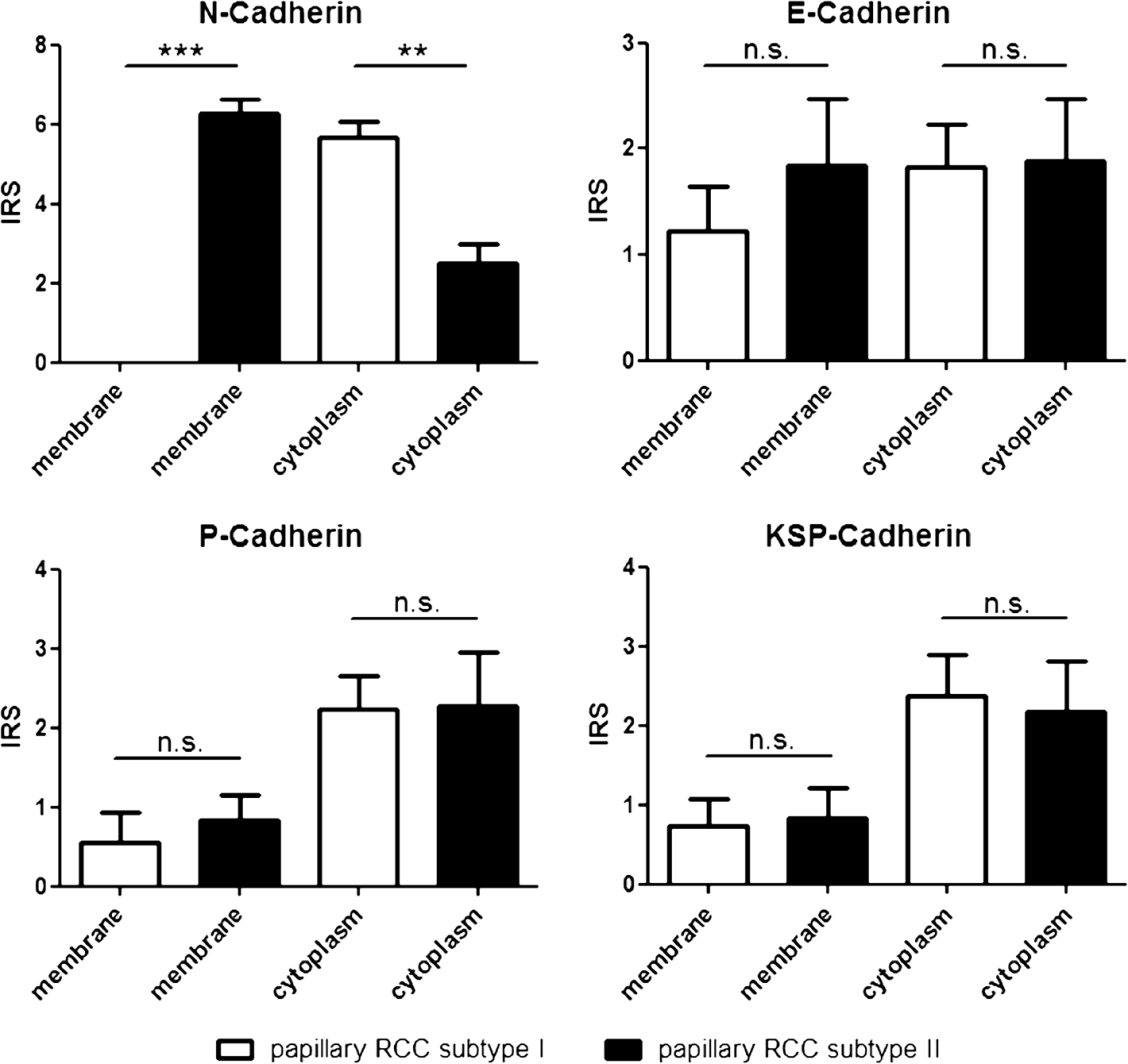
**Immunohistochemistry of E-, P-, KSP- and cadherin expression in papillary RCC.** Expression of E- (**A** / **D**), P- (**B** / **E**) and KSP-cadherin (**C** / **F**) in papillary RCC type II (**A**, **B**, **C** x20) and type I (**D**, **E**, **F** x20). The expression patterns are not suitable for a clear cut differentiation of both tumor subtypes (for details see results).

The E-cadherin staining showed in the majority of papillary RCC type II a weak membranous (IRS 1.83 ± 2.72) as well as cytoplasmic expression (IRS 1.89 ± 2.49). In papillary RCC type I a decreased expression of membranous (IRS 1.28 ± 1.95) and an approximately similar cytoplasmic E-cadherin (IRS 1.81 ± 1.94) could be determined. A statistical comparison showed no significant difference for membranous as well as cytoplasmic E-cadherin expression in both subtypes of papillary RCC (p = n. s.) (Figure 3A, D and Figure 2).

A weak membranous expression of P-cadherin could be demonstrated for type I (IRS 0.54 ± 1.79) and type II (IRS 0.83 ± 1.38) papillary RCC without significant differences (p = n .s.). The investigation of cytoplasmic P-cadherin demonstrated an almost equal expression in type II and type I papillary RCC (IRS 2.27 ± 2.86 vs. 2.22 ± 1.97) (Figure 3B, E and Figure 2).

A membranous KSP-cadherin expression could only be demonstrated in some cases of papillary RCC type II (IRS 0.83 ± 1.65) and papillary RCC type I (IRS 0.73 ± 1.61). Cytoplasmic KSP-cadherin was only weakly expressed in papillary RCC type II (IRS 2.17 ± 2.71) as well as type I (IRS 2.36 ± 2.44). The statistical analysis showed no significant differences for membranous and cytoplasmic KSP-cadherin expression in both subtypes of papillary RCC (p = n. s.) (Figure 3C, F and Figure 2).

A correlation of cadherin expression with tumor grade or tumor stage could not be observed.

Normal kidney tissue showed an irregular weak to intermediate cytoplasmic expression of the investigated cadherins in proximal and distal tubular. A membranous expression of cadherins could not be observed at all.

## Discussion

Papillary RCC represents a subtype of RCC with a typical morphology and typical characteristic genetic aberrations [3,6]. Several investigations could define a prognostic relevant subdivision into type I and type II papillary RCC [7-9]. This subdivision can so far only be done by morphological criteria [16,17]. Several immunohistochemical markers were applied to find any useful differentiation criteria between the two subtypes. Cytokeratin-7 could be etablished as a helpful marker, because it is detectable in more than 80% of type I tumors, whereas type II papillary RCC in only 20% express cytokeratin-7 [3,18]. Perret et al. could demonstrate a significant higher MUC-1 expression in type I papillary RCC [19]. Zhou et al. [20] described that E-cadherin could help to distinguish between type I and type II of papillary RCC. Other typical antigens for renal neoplasm such as CD10 were also investigated but did not show any significant differences [21,22]. N-cadherin, primarily described as A-CAM, was shown to be expressed by normal renal epithelium [23,24]. Furthermore Markovic-Lipovski et al. could show an expression of N-cadherin in different types of RCC, but they did not analyse papillary RCC [25].

We investigated the expression of four different cadherins in type I and type II papillary RCC. It could be demonstrated that all investigated papillary RCC type II showed a membranous expression of N-cadherin, whereas type I did not show any membranous N-cadherin. In contrast, type I papillary RCC showed a significant higher cytoplasmic expression for N-cadherin compared to type II. Cadherins are transmembrane glycoproteins, which act as cell-cell contacts and in signal transduction. Only if the cadherins are localized in the membrane, these functions can be executed [26]. Therefore the observed cytoplasmic cadherin expression must be accompanied by a loss function. N-cadherin is normally expressed in neuronal tissue and plays a key role in organ development [27]. An non tissue specific expression of N-cadherin as found for papillary RCC type II could be shown to induce cell migration, metastases, and invasion especially in breast cancer [14]. This process is also known as epithelial-mesenchymal transition (EMT) and it plays a crucial role in embryonic development [28]. In tumors this transition promotes the mobility and invasive capacity of tumor cells and it is associated with a progression of tumor disease [29,30]. In addition EMT is connected with cancer stem cell-like features, which include the development of resistances to chemotherapy [31,32].

These observations are well in line with the worse behaviour of papillary RCC type II in comparison to type I as previously described [7-9]. For different other tumors it could also be shown that not only the non tissue specific expression of N-cadherin but also the switch from different cadherins to N-cadherin is associated with a worse prognosis [33,34]. In addition the activation of the PI-3 kinase / Akt Pathway induced by N-cadherin could be demonstrated as a survival mechanism for lung cancer [35].

Our data show a higher expression of E-cadherin in papillary RCC type II. It is widely accepted that an increased expression of E-cadherin in cancer is associated with a better outcome and a decrease occurrence of metastases [13]; in ovarian carcinoma lower E-cadherin seems to influence the transition from normal ovarian surface epithelium to ovarian cancer [36]. The investigation of KSP-cadherin showed only a weak membranous and cytoplasmic expression in both subtypes of papillary RCC. These findings for KSP-cadherin correlate with those of other working groups [37]. P-cadherin expression which plays an important role in ovarian cancer also in case of cadherin switch [38] did not show a prominent expression in papillary RCC.

In conclusion, N–cadherin could be established as the first immunohistological marker for a clear cut differentiation between papillary RCC subtype I and II. Furthermore, the data implicate that cadherins especially N-cadherin and the involved pathway via p120 catenin, which is used by most cadherins, could play a pivotal role for the therapy of RCC as already shown for other neoplasias [39-41].

## Competing interests

The authors declare that they have no competing interests.

## Authors’ contributions

CLB constructed the manuscript and carried out pathological examination. BH participated in pathological investigations. AS was responsible for the clinical data. HJR and FB were responsible for critical revision of the manuscript and have been involved in drafting it. All authors read and approved the final manuscript.
